# RETRACTED: Abdullah et al. New Amphiphilic Ionic Liquids for the Demulsification of Water-in-Heavy Crude Oil Emulsion. *Molecules* 2022, *27*, 3238

**DOI:** 10.3390/molecules31152555

**Published:** 2026-07-23

**Authors:** Mahmood M. S. Abdullah, Abdelrahman O. Ezzat, Hamad A. Al-Lohedan, Ali Aldalbahi, Ayman M. Atta

**Affiliations:** 1 Department of Chemistry, College of Science, King Saud University, P.O. Box 2455, Riyadh 11451, Saudi Arabia; maltaiar@ksu.edu.sa (M.M.S.A.); hlohedan@ksu.edu.sa (H.A.A.-L.); aaldalbahi@ksu.edu.sa (A.A.); 2 Petroleum Application Department, Egyptian Petroleum Research Institute, Cairo 11727, Egypt

The Journal retracts the article “New Amphiphilic Ionic Liquids for the Demulsification of Water-in-Heavy Crude Oil Emulsion” [1].

Following publication, concerns were brought to the attention of the Editorial Office regarding the integrity of the figures presented in this article.

In accordance with the journal’s standard procedures, the Editorial Office and Editorial Board conducted an investigation, which identified indications of inappropriate image editing and duplication in Figures 2 and 5. Although the authors cooperated with the investigation, the explanations and supporting materials provided were not deemed sufficient by the Editorial Board to resolve the identified concerns.

As a consequence, the Editorial Board has lost confidence in the validity of the images and has decided to retract this publication in accordance with MDPI’s retraction policy.

This retraction was approved by the Editor-in-Chief of the journal *Molecules*.

The authors have been informed of this retraction but did not provide a comment on this decision.
